# The Emerging Role of Protein Phosphatase in Regeneration

**DOI:** 10.3390/life13051216

**Published:** 2023-05-19

**Authors:** Meiling Zhang, Chenglin Liu, Long Zhao, Xuejiao Zhang, Ying Su

**Affiliations:** 1 Institute of Evolution & Marine Biodiversity, Ocean University of China, Qingdao 266003, China; 2 College of Fisheries, Ocean University of China, Qingdao 266003, China; 3 College of Marine Life Sciences, Ocean University of China, Qingdao 266003, China

**Keywords:** protein phosphatase, phosphorylation, regeneration, organ

## Abstract

Maintaining normal cellular behavior is essential for the survival of organisms. One of the main mechanisms to control cellular behavior is protein phosphorylation. The process of protein phosphorylation is reversible under the regulation of protein kinases and protein phosphatases. The importance of kinases in numerous cellular processes has been well recognized. In recent years, protein phosphatases have also been demonstrated to function actively and specifically in various cellular processes and thus have gained more and more attention from researchers. In the animal kingdom, regeneration frequently occurs to replace or repair damaged or missing tissues. Emerging evidence has revealed that protein phosphatases are crucial for organ regeneration. In this review, after providing a brief overview of the classification of protein phosphatases and their functions in several representative developmental processes, we highlight the critical roles that protein phosphatases play in organ regeneration by summarizing the most recent research on the function and underlying mechanism of protein phosphatase in the regeneration of the liver, bone, neuron, and heart in vertebrates.

## 1. Introduction

Proteins could gain functional maturity through post-translational modifications (PTMs), including the covalent addition of an active group, proteolytic processing, and necessary folding. PTMs include many modifications, such as methylation, acetylation, and phosphorylation. The study of phosphorylation is extensive and related to many regulating processes of cellular behavior, including cell growth, differentiation, and apoptosis [1]. The occurrence of phosphorylation is widespread in organisms, and around 30% of proteins could be phosphorylated in eukaryotes [2]. The phosphorylation sites are mainly on threonine, serine, and tyrosine, which are all hydroxyl-containing amino acids [3]. Proteomic analysis of 2244 human proteins in Hela cells has indicated that the proportion of phosphorylation on serine, threonine, and tyrosine residues accounts for 86.4%, 11.8%, and 1.8%, respectively [4]. After a protein is phosphorylated, its conformation may change, and its affinity to another protein may be affected, resulting in the activation or inactivation of this protein.

Protein phosphorylation is a dynamic and reversible process regulated by two types of enzymes, protein kinase and protein phosphatase. Protein kinases transfer the phosphate groups from ATP to serine, threonine, or tyrosine residues on substrate proteins. The phosphate group is extracted from the substrate proteins by the protein phosphatase [5]. Protein phosphatases were initially considered to act merely as a passive and unspecific negative regulator of phosphorylation, so they gained much less attention than protein kinases for a long time. In recent decades, more and more evidence has shown that protein phosphatases play active and selective roles in various fields, and therefore there has been much more focus on them than in the past [5,6,7].

Recently, the important role of protein phosphatases in the regeneration process has been gradually realized. Regeneration occurs widely in the animal kingdom and is commonly defined as replacing the injured body part with the newly generated tissue with the same morphology and function. This process involves the formation of a wound epithelium after injury, the generation of regenerative progenitor cells, and morphogenesis [8,9]. The capacity for regeneration differs greatly across organs and organisms [10]. Increasingly studies have demonstrated that the manipulation of the stability or activity of certain protein phosphatases is able to alter the regenerative ability of tissues/organs. In this review, we summarize the recent research progress regarding the crucial functions of protein phosphatases in the regeneration of several critical vertebrate organs. 

## 2. Classification of Protein Phosphatases

Based on the specificity of substrate proteins, protein phosphatases in eukaryotes can be divided into the protein tyrosine phosphatase family (PTP) and protein serine/threonine phosphatase family (PSTP) [5,11,12]. The classification of protein phosphatases is listed in detail in Table 1.

PTPs that dephosphorylate phosphotyrosine (pTyr) have been classified into five classes (I-V), based on the amino acid sequence within their catalytic domains and the nucleophilic amino acid used during the catalytic reaction. The PTPs of Classes I, II, and III are cysteine-based phosphatases, and their catalytic domains have the signature motif cysteine-X5-arginine (where X can be any amino acid). In contrast, Class IV comprises aspartate-based PTPs that utilize a different catalytic mechanism and have a key aspartate in the active site of the enzyme [11,13]. Class V contains histidine-based phosphatases [14]. Among these five classes, Class I is the largest group including classical PTPs (strictly tyrosine-specific) and dual specificity phosphatases (DUSPs, also known as DSPs). The classical PTPs are further divided into two groups: the transmembrane, receptor-like PTPs (RPTPs), and the intracellular, non-receptor-like PTPs (NRPTPs) [13,15,16]. DUSPs have a much broader range in terms of substrate specificity. Their targets could be pTyr, phosphoserine (pSer), phosphothreonine (pThr), and phosphoinositide (PIP). PTEN (phosphatase and tensin homolog deleted on chromosome 10), a member of DUSP, is a well-known lipid phosphatase dephosphorylating the D3-phosphate of inositol phospholipids [16]. Class II comprises only one member, low molecular weight phosphatase (LMW-PTP), which acts on signaling transduction pathways induced by growth factors and cytokines [17]. Class III PTPs refer to cell division cycle 25 (CDC25) phosphatases, which are involved in cell cycle regulation. Eya (Eyes absent) multifunctional proteins belong to Class IV [13,16,18,19,20]. ClassV includes Sts-1 (suppressor of T-cell receptor signaling-1) and Sts-2, which are crucial regulators of the T cell receptor (TCR) signaling [14,21].

PSTPs dephosphorylate pSer or pThr, and are classified into at least three families, including serine/threonine-specific phosphoprotein phosphatases (PPPs), metal-dependent protein phosphatases (PPMs), and aspartate-based protein phosphatases (DxDxTs) [22]. PPPs play significant roles in many important cellular signaling pathways related to cell division and growth. More than 90% of serine/threonine dephosphorylations depend on PPPs [23]. Seven different PPPs have been found in the human genome, including protein phosphatase 1 (PP1), PP2A, PP2B (also known as PP3 or calcineurin), PP4, PP5, PP6, and PP7. They mostly function as a multimeric holoenzyme, composed of the catalytic and regulatory subunits, to dephosphorylate different substrates. PP1 and PP2A are the two most abundant protein phosphatases in cells [22,24]. PPMs are Mn^2+^/Mg^2+^-dependent serine/threonine-specific enzymes, including PP2C and heterodimeric pyruvate dehydrogenase phosphatases (PDPs) [18,25]. The aspartate-based protein phosphatases rely on the aspartic acids of the sequence motif DxDxT/V to obtain phosphatase activity [11,18].

**Table 1:** Classification of protein phosphatases based on publications.

Superfamily	Family	Subfamily	Reference
PTPs	Class I	RPTPs	[16,17]
		NRPTPs	
		DUSPs	
	Class II	LMW-PTP	[17]
	Class III	CDC25	[17,19]
	Class IV	Eya	[17,20]
	Class V	Sts	[14]
PSTPs	PPPs	PP1	[22,24]
		PP2A	
		PP2B	
		PP4	
		PP5	
		PP6	
		PP7	
	PPMs	PP2C	[18,22,25]
		PDP	
	DxDxTs	FCP/SCP	[18,22]
		HAD	

Abbreviations: RPTP, receptor-like protein tyrosine phosphatase; NRPTP, non-receptor-like protein tyrosine phosphatase; DUSP, VH1-like dual specificity phosphatase; LMW-PTP, low molecular weight protein tyrosine phosphatase; CDC25, cell division cycle 25; Eya, Eyes absent; Sts, suppressor of T-cell receptor signaling; PDP, pyruvate dehydrogenase phosphatase; FCP/SCP, TFIIF (transcription initiation factor IIF)-associating component of CTD (C-terminal domain) phosphatase/small CTD phosphatase; HAD, haloacid dehalogenase family enzyme.

## 3. Various Developmental Functions of Protein Phosphatase

**Figure 1:**
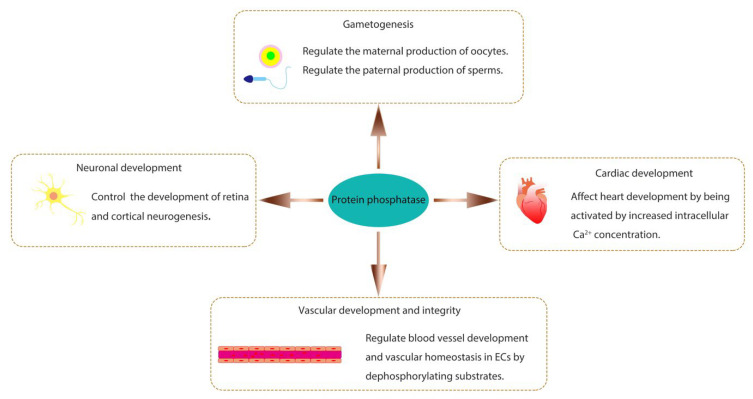
Various biological functions of protein phosphatase.

Protein phosphatases are related to numerous biological processes and play important roles in organisms. Several well-studied biological functions of protein phosphatases during animal development are briefly introduced here (Figure 1).

Protein phosphatases are essential for gametogenesis. Protein kinase and phosphatase signaling pathways can regulate both the maternal production of oocytes and the paternal production of sperms [26]. The type 2A protein phosphatase subfamily, which includes PP2A, PP4, and PP6, has been implicated to play critical roles in regulating germ cell meiosis [27]. In this subfamily, PP2A is well-known and extensively studied. In *Xenopus* oocytes, it has been demonstrated that PP2A acts on Arpp19 to initiate meiotic division and regulate M-phase entry by antagonizing the activities of two kinases, cAMP-dependent protein kinase A (PKA) and Greatwall (Gwl) [28]. The serine 109 (S109) on Arpp19 can be phosphorylated by PKA but dephosphorylated by PP2A containing B55δ regulatory subunit (PP2A-B55δ). The balance between these two opposite events maintains the prophase arrest of oocytes to allow their growth and nutrient accumulation. Upon hormonal stimulation by progesterone, PKA activity is downregulated due to a reduced cAMP level. In contrast, PP2A activity is not altered, resulting in the dephosphorylation of S109 on Arpp19, which releases oocytes from prophase arrest and initiates meiotic division. Consequently, the phosphorylation of Arpp19 at serine 67 (S67) by Gwl can inhibit PP2A activity and fully activate Cdk1, triggering the M-phase entry [28]. The involvement of PP6 in gametogenesis has also been reported recently. In male mice, PP6 regulates meiotic recombination and fertility. The loss of PP6 in germ cells causes abnormal MAPK pathway activity, which affects chromatin relaxation. Ultimately, programmed double-stranded break (DSB) repair factors are prevented from being recruited to appropriate sites on the chromosome, and the spermatocytes are arrested at the pachytene stage during the meiotic process [29]. In female mice, PP6 is dispensable for oocyte meiotic maturation but essential for their meiosis II exit. The loss of PP6 in oocytes also causes the impaired fertility of mice [30]. In addition to PP2A and PP6, PP4 is also essential for normal sperm production. The deletion of PP4 catalytic subunit gene in the mouse germ cells causes sperm tail-bending defects, low sperm count, and poor sperm motility, resulting in male infertility [31].

The role of protein phosphatase has also been studied in cardiac development. Calcineurin (PP2B), a Ca^2+^-dependent PSTP, is crucial for cardiomyocytes through dephosphorylating the transcription factor nuclear factor of activated T cells (NFAT) in the cytoplasm, which subsequently undergoes nuclear translocation to regulate gene expression [32,33,34]. It has been found that PPP2R3A, one of the PP2A regulatory subunits, is required for normal myocardium formation and efficient cardiac contractile function in zebrafish [35,36]. PPP2R3A has two transcripts, *pr72* and *pr130*. The zebrafish with *pr72*-deletion or *pr130*-knockout exhibits cardiac developmental abnormalities, including reduced cardiomyocytes, abnormal ventricular chambers, cardiac looping defects, and decreased cardiac function [35,36]. The phosphatase Pez, a member of PTP, is expressed transiently in the heart of zebrafish embryos. Its knockdown causes a heart looping defect and the lack of functional atrio-ventricular (A-V) valves [37]. 

Protein phosphatases are also involved in the growth and integrity of the vascular system. Vascular endothelial protein tyrosine phosphatase (VE-PTP, or PTPRB), a receptor-type phosphatase, is predominantly expressed in vascular endothelial cells and is crucial for angiogenesis during development [38,39]. It regulates vascular integrity by dephosphorylating substrates that control endothelial junctions, such as the endothelial adhesion molecule VE-cadherin, the angiopoietin receptor TIE2, and the vascular endothelial growth factor receptor VEGFR2 [39,40]. Mouse embryos with disruption of the VE-PTP gene have severe vascular malformations, including a disorganized brain vascular network and the loss of intersomitic vessels, causing early lethality [41]. Additionally, PP2A is also a significant regulator of angiogenesis. PP2A-Bα, a regulatory subunit of PP2A, is required for vascular lumen integrity in zebrafish by controlling the phosphorylation status and activity of histone deacetylase 7 (HDAC7), an essential transcriptional regulator of vascular stability [42]. PP2A also regulates angiogenesis by mediating the activity of the Hippo signaling pathway effector Yes-associated protein (YAP), which can promote endothelial cell proliferation, migration, and sprouting in mice [43].

The importance of protein phosphatases also cannot be ignored for neuronal development. Neurons are polarized cells composed of a cell body, a single axon, and dendrites [44,45]. The receptor-type phosphatase family members, such as PTPα, PTPγ, PTPδ, and PTPσ, have been implicated in playing diverse roles throughout the neural development in vertebrates and invertebrates, regulating neurogenesis, axon growth and guidance, synapse formation and plasticity [46,47]. The dual specificity phosphatase DUSP26 is specifically expressed in neuroendocrine tissues and has been reported to phosphorylate nerve growth factor (NGF) receptor TrkA and fibroblast growth factor receptor 1 (FGFR1) to maintain proper development of the retina and neuronal system in zebrafish [48].

During bone formation, mesenchymal stem cells (MSCs) differentiate into osteoblasts and osteocytes. BMP signaling is well recognized as an essential inducer of this process [49]. Various protein phosphatases have been reported to regulate MSC differentiation through the BMP signaling pathway. BMP signaling is activated by the phosphorylation of Smad proteins. PPM1A/PP2Cα, one of the serine/threonine phosphatases in the PPM family, has been shown to suppress BMP signaling by dephosphorylating Smad proteins [50]. Differently, a novel mechanism has also been reported in which PPM1A regulates the protein levels of Smads via the proteasome pathway, thus affecting BMP signaling activities [51]. In this study, the knockdown of endogenous PPM1A stimulates the differentiation of osteoblasts [51]. Moreover, PP2Acα negatively regulates the phosphorylation of Smad1/5/9, inhibiting the BMP2-induced osteoblast differentiation [52]. The SCP family of nuclear phosphatases, as a type of Smad phosphatase in the nucleus, has been proven to control BMP signaling and regulate MSC differentiation by mediating Smad1/5/8 dephosphorylation [53,54]. In addition to PSTPs, DUSPs have been found to activate BMP-Smad1 signaling and promote osteogenic differentiation of MSCs by reducing the SCP-Smad1 interaction [54], and the histidine phosphatase Sts-1 can regulate bone remodeling by modulating osteoclast function [55]. 

Overall, emerging evidence has demonstrated that protein phosphatases play important and diverse roles in a wide range of biological processes, including but not limited to the events mentioned above. Next, we will focus on the regeneration process to highlight the role of protein phosphatases in it.

## 4. Roles of Protein Phosphatases in Regeneration

Regeneration is commonly defined as the structural and functional recovery of injured organs or lost body parts [10]. The demands for regenerative medicines in clinics largely foster tissue regeneration studies to explore what factors control proliferation and patterning during regeneration. Increasing research over the last few decades has shown that protein phosphatase is a crucial regulator for organ regeneration.

### 4.1. Liver Regeneration

The liver is an important organ in the body for metabolic regulation. It can regenerate after resection or injury, in which hepatocytes fully differentiate and re-enter the cell cycle to divide and proliferate [56]. The classic regeneration process consists of three stages, initiation, proliferation, and termination [57]. The most commonly used experimental model for investigating liver regeneration in rodents is the partial hepatectomy (PH) [58]. Protein phosphatases have been involved in the control of hepatocyte proliferation and body homeostasis after PH.

PTP1B, as a non-receptor PTP, participates in metabolic and liver diseases [59]. Recent studies have shown that PTP1B plays a fundamental role in liver regeneration after PH in mice [60,61]. In the initiation stage after liver damage, PTP1B-deficient mice trigger more rapid mitogenesis by accelerating the phosphorylation of JNK1/2 and STAT3 mediated by TNF-α and IL-6 [60]. During hepatocyte proliferation, more intrahepatic lipids are accumulated to provide energy fuel in PTP1B knockout mice through enhanced EGF- and HGF-mediated AKT and ERK signaling [60,61]. In the termination phase of liver regeneration, the growth factor- and cytokine-mediated proliferative signalings are inhibited to control liver size. PTP1B deficiency delays the termination of liver regeneration by inhibiting the transforming growth factor β (TGF-β) signaling, the main antiproliferative factor within the liver [60].

Along with PTP1B, PP2A also affects the termination of liver regeneration. The catalytic subunit of PP2A, PP2Acα, has already been shown in numerous studies to be essential for cell cycle control [62]. The deletion of PP2Acα, specifically in hepatocytes in mice, can accelerate hepatocyte proliferation by activating AKT, which in turn inhibits the activity of glycogen synthase kinase 3β (GSK3β) and leads to the accumulation of cyclin D1 protein in hepatocytes, resulting in delayed termination of liver regeneration [63]. Furthermore, the liver-specific knockout of PP2Acα activates PFKFB2, an isoform of the key glycolytic enzyme 6-phosphofructo-2-kinase/fructose-2,6-bisphosphatase (PFKFB), increasing the hepatocyte glycolysis and delaying the termination of liver regeneration [64]. 

Protein phosphatases also act on the Hippo signaling pathway to regulate hepatocyte proliferation, damage response, and liver size [65]. The mammalian sterile 20-like kinase 1 and 2 (MST1/2) and large tumor suppressor 1 and 2 (LATS1/2) make up the core of the Hippo signaling cascade, which phosphorylates downstream effectors YAP and transcriptional co-activator TAZ to keep them in the cytoplasm [66]. In the mouse liver, deletion of Mst1/2 results in the hyperproliferation of hepatocytes [67,68]. By antagonizing the activities of kinases, several protein phosphatases have been reported to regulate the Hippo-YAP pathway, including PP1 that selectively dephosphorylates TAZ and LATS1 [69,70], and PP2A that targets YAP and regulates mammalian epidermal maintenance [71]. The phosphatase PTPN14 interacts with YAP and promotes its cytoplasmic translocation. However, this process is independent of PTPN14 phosphatase activity [72,73]. Furthermore, PPM1A directly eliminates YAP phosphorylation at the critical S127 residue, which drives YAP/TAZ accumulation in the nucleus to activate target gene expression [74]. In the PPM1A knockout mice with hepatectomy surgery, the injury-induced compensatory hepatocyte proliferation is down-regulated and liver regeneration is compromised [74], indicating that PPM1A is a critical YAP phosphatase to facilitate liver regeneration. 

**Figure 2:**
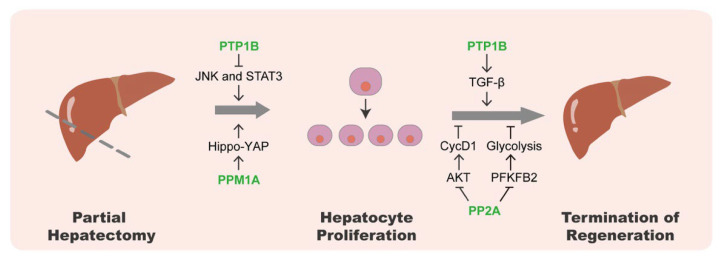
The roles of protein phosphatases in liver regeneration. During liver regeneration, PTP1B inhibits hepatocyte proliferation through the JNK and STAT3 signaling pathways, and is also required for TGF-β signaling to promote the termination of regeneration. PPM1A stimulates hepatocyte proliferation through the Hippo-YAP signaling pathway. In the termination stage of liver regeneration, PP2A enhances hepatocyte proliferation and delays the termination of regeneration through AKT-Cyclin D1 and PFKFB2-glycolysis pathways.

Collectively, multiple protein phosphatases play important roles in regulating liver regeneration by controlling hepatocyte proliferation and the termination time point of regeneration (Figure 2).

### 4.2. Nerve Regeneration

It is well known that the human nervous system includes the central nervous system (CNS) and the peripheral nervous system (PNS). They are composed of various nerve cells, including neurons, glial cells, Schwann cells, and astrocytes, which coordinate locomotion, sensory perception, and homeostasis in animals [75]. However, CNS in higher organisms has limited regenerative capacity after physical damage or disease such as stroke, which deeply alters the lives of affected individuals and leads to disability and death [76]. Therefore, exploring the endogenous mechanisms to stimulate the regeneration of the CNS will help develop novel therapeutic measures.

**Figure 3:**
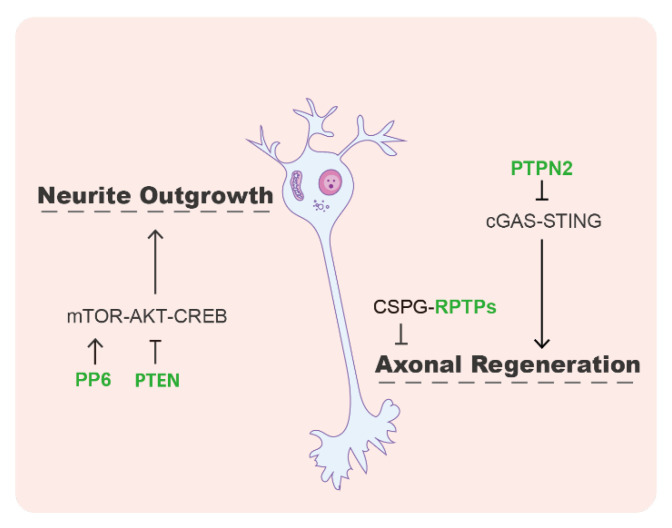
The roles of protein phosphatases in neural regeneration. PP6 promotes, but PTEN inhibits, neural regeneration through the mTOR/AKT/CREB signaling pathway. The receptor-type PTPs (RPTPs) act as the receptor of CSPG that has an inhibitory effect on axon growth and regeneration. PTPN2, a non-receptor PTP, hinders axon regeneration by suppressing the cGAS-STING pathway.

Nerve regeneration refers to generating new neurons or restoring the neuronal structure. Studies have shown that protein phosphatases play an important role in axon growth and neuronal regeneration by acting on a variety of key factors and signaling pathways (Figure 3). The receptor-type phosphatase PTPσ has been reported to mediate neuronal regeneration and development [77]. PTPσ contains a cell adhesion molecule-like extracellular region and triggers signals in response to cell-cell or cell–extracellular matrix contacts [78]. PTPσ has been identified as a major receptor for chondroitin sulfate proteoglycans (CSPGs), which exhibits an inhibitory effect for neuronal repair [77,79]. After brain or spinal cord injury, CSPGs accumulate in pathological scars, inhibiting axonal growth and neural regeneration [77]. Modulation of PTPσ by a synthetic intracellular sigma peptide (ISP, as a PTP inhibitory peptide) could enhance the degradation of CSPGs and thus promote axon outgrowth [80]. In the stroke mouse model, ISP treatment or PTP deletion improves stroke recovery, along with neuroprotection, axonal sprouting, and migration of new neuroblasts [81]. Another phosphatase in the subfamily that PTPσ belongs to, leukocyte common antigen-related phosphatase (LAR), was also identified as a functional receptor of CSPGs to inhibit axon growth [82].

In addition to the receptor-like PTPs, several other phosphatases were also reported to regulate axon regeneration. It has been known that the mammalian target of rapamycin (mTOR) phosphorylates AKT, then AKT phosphorylates the cAMP response element binding protein (CREB), which is one of the major transcriptional factors positively regulating neurite outgrowth [83]. The lipid phosphatase PTEN acts as a negative regulator of the mTOR/AKT/CREB signaling pathway. Deletion of PTEN can promote axon regeneration after nerve injury [84]. PP6 was discovered to promote neurite growth by dephosphorylating SIN1, a component of mTOR complex 2. The dephosphorylated form of SIN1 can facilitate the mTOR-mediated AKT phosphorylation and downstream CREB signaling [83]. In addition, a recent study showed an mTOR-independent mechanism used by protein tyrosine phosphatase non-receptor type 2 (PTPN2) to control axonal regeneration. In PTPN2 knockout mice, the DNA-damage-induced cGAMP synthase (cGAS)-stimulator of interferon genes (STING) pathway is activated and triggers the expression of interferon-stimulated genes (ISGs) in neurons, which ultimately promotes axon regeneration in the central nervous system [85].

### 4.3. Heart Regeneration

The irreversible loss of heart muscle cells caused by heart diseases, such as myocardial infarction (MI or heart attack), leads to heart failure [86]. Stimulating myocardial regeneration would be a promising therapeutic strategy to reduce morbidity and mortality of heart diseases. The regenerative capacity of the heart varies considerably among species [87]. Adult mammals with a poor rate of cardiomyocyte turnover cannot regain their original structure and function after an external lesion or disease of the heart, but instead, form permanent scars of massive fibrous tissue at the wound site [88]. It is interesting to note that the hearts of neonatal mammals, including mice or pigs, possess a certain capacity to repair damage during the first week or the first two days of life [89,90]. Surprisingly, adult zebrafish can fully regenerate their heart after amputating up to ∼20% of the ventricle [91]. The meticulous and profound studies of cardiac regeneration in animal models may lead to new development in clinical therapies that benefit millions of people annually.

**Figure 4:**
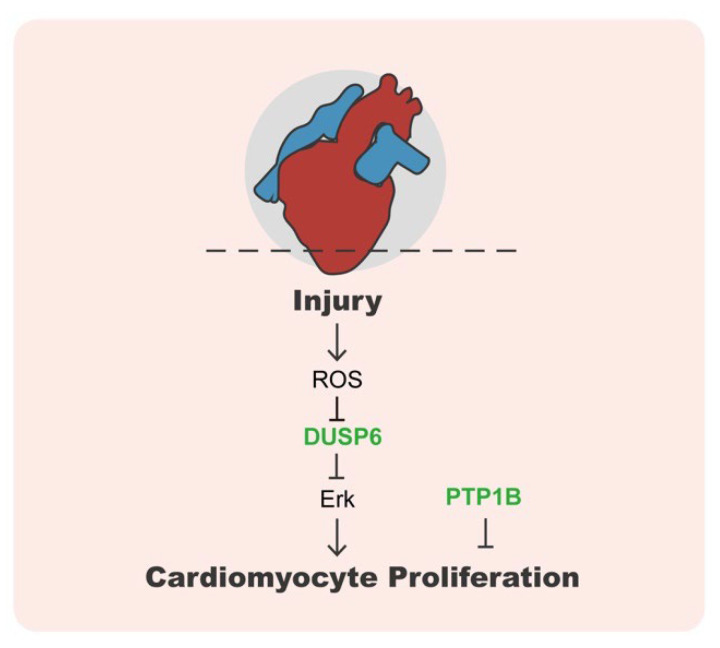
The roles of protein phosphatases in heart regeneration. Upon heart injury, ROS at the wound site inhibits DUSP6 and promotes cardiomyocyte proliferation. In addition, inhibition of PTP1B with the small molecule MSI-1436 can promote cardiomyocyte proliferation and improve the recovery of cardiac function.

During zebrafish heart regeneration, the continuous increase in reactive oxygen species (ROS) can promote the regeneration response. ROS exists in multiple forms and is defined as highly reactive ions and free radicals in the form of hydrogen peroxide (H_2_O_2_), superoxide anion (O^2−^), and hydroxyl radical (OH^−^) [92]. The recent finding in zebrafish showed that DUSP6 is a potential downstream target of ROS and can effectively regulate cardiac regeneration (Figure 4) [92]. DUSP6 is a well-known phosphatase that specifically dephosphorylates extracellular signal-regulated kinase 1/2 (ERK1/2), therefore acting as an attenuator of Ras/MAPK signaling. DUSP6 is also sensitive to redox. H_2_O_2_ produced after heart injury can destabilize DUSP6 and increase the phosphorylation of ERK1/2 and Ras/MAPK signaling activity [93]. Consistently, suppressing DUSP6 function in zebrafish promotes cardiomyocyte proliferation and coronary angiogenesis, but reduces fibrosis after the ventricular resection [94]. Furthermore, DUSP6 deficiency in rats and mice improves cardiac repair and function by balancing p38 and pERK activity and, ultimately, reducing neutrophil-mediated cell death and tissue damage [95,96]. Another member of DUSPs, PTEN was also found to regulate cardiac repair after MI [97]. PTEN deficiency in mice directly promotes cardiomyocyte proliferation via regulating PI3K/AKT signaling to enhance myocardial repair in response to MI [98]. All these animal studies provide potent therapeutic targets for cardiac remodeling after MI and other related diseases.

Moreover, DUSPs have been reported to modulate heart functions in other aspects. DUSP1 functions as an anti-inflammatory factor in cardiovascular disorders. DUSP1 overexpression can attenuate inflammation-induced myocardial injury by improving mitophagy and mitochondrial metabolism in the mice model of the septic cardiomyopathy [99]. DUSP26 was found to promote aortic valve calcification. DUSP26 is up-regulated in calcific aortic valve disease (CAVD), and its silencing can reduce aortic valve calcification in mice model [100]. Although these studies are not directly related to regeneration, these phosphatases could be of potential significance in cardiac regeneration, as inflammation response and fine structure reconstruction should be fully considered for complete organ regeneration.

In addition to DUSPs, inhibition of PTP1B with small molecule MSI-1436 is also able to promote cardiomyocyte proliferation and improve the recovery of cardiac function [101]. MSI-1436 treatment accelerates heart regeneration in adult zebrafish by promoting cardiomyocyte proliferation. In addition, MSI-1436 treatment in adult mice with coronary artery ligation can improve cardiac function, reduce infarct size, and increase cell proliferation in the infarct border zone [101]. Thus, PTP1B could be a new and promising therapeutic target for treating heart disease and stimulating cardiac regeneration (Figure 4).

### 4.4. The Regeneration of Other Organs or Cells

In addition to the organs or tissues mentioned above, protein phosphatases are also involved in zebrafish fin regeneration. Zebrafish fins are well-organized structures that can entirely regenerate throughout their lives, which is accomplished by forming regenerative blastema at the wound site [102]. Several factors, including calcineurin, are required for fin regeneration. Calcineurin is associated with the inhibition of retinoic acid signaling and modulates the isometric and allometric coordinated growth of developing and regenerating zebrafish fins to establish an appropriate size [103]. Moreover, the cooperation of calcineurin activity and retinoic acid signaling activity regulates the blastema cell differentiation toward joint cells and osteoblasts in regenerating fins [104]. Interestingly, recent studies have shown that calcineurin controls proximodistal blastema polarity in zebrafish fin regeneration [8].

A recent study indicated that PTPs are important in hematopoietic stem cell (HSC) regeneration. HSCs regulate their own maintenance, proliferation, and differentiation, by coordinating several receptor tyrosine kinases (RTKs) and PTPs. Recently, it was discovered that HSCs express PTPσ, a phosphatase primarily expressed by neurons. Treatment with PTPσ inhibitors in irradiated mice can promote HSC regeneration, accelerate hematologic recovery, and improve survival [105].

## 5. Conclusions and Perspective

Reversible phosphorylation controlled by protein kinases and protein phosphatases constitutes a major form of signaling in all living organisms. In vivo, a variety of protein phosphatases play an essential role in many biological processes. In this review, we primarily discussed a series of important roles that protein phosphatases play in the regeneration of several organs. Protein phosphatases regulate many important signaling pathways during regeneration, including the JNK, STAT, TGF-β, and Hippo signaling pathways. Accumulating evidence supports the idea that regulating the protein level or activity of protein phosphatase could be an effective way to modulate the ability and rate of regeneration. 

Recently, small molecular medicine targeting protein phosphatases has become an emerging regenerative tool to promote the repair and regeneration of injured tissues. Studies have shown that different miRNAs can regulate phosphatases, including miR-222, which targets PTEN and enhances neuronal regrowth after injury [106], and miR-26a-5p, which regulates the PTEN/AKT signaling pathway and protects against myocardial ischemia/reperfusion injury [107]. Additionally, the development and application of the affinity-directed phosphatase (AdPhosphatase) system enable targeted dephosphorylation of specific phospho-substrates [108]. This study also indicates that nanotechnology could be an effective way to deliver phosphatase regulators into damaged sites to promote regeneration. Nevertheless, the precise targets and mechanisms of protein phosphatases during regeneration need to be further explored before the clinical application of this type of medicine.

In a word, exploring the molecular mechanisms of protein phosphatases in organ regeneration will improve understanding of the molecular regulatory network of regeneration and provide new insights for clinical strategies to activate the regenerative potential of mammals, including humans.

## Data Availability

Not applicable.
